# 4-Iminooxazolidin-2-One as a Bioisostere of Cyanohydrin Suppresses EV71 Proliferation by Targeting 3C^pro^

**DOI:** 10.1128/Spectrum.01025-21

**Published:** 2021-11-17

**Authors:** Binghong Xu, Meijun Liu, Sen Ma, Yuying Ma, Si Liu, Luqing Shang, Cheng Zhu, Sheng Ye, Yaxin Wang

**Affiliations:** a School of Life Sciences, Tianjin University, Tianjin, People’s Republic of China; b College of Pharmacy, Nankai University, Tianjin, People’s Republic of China; c Department of Chemistry, Texas A&M University, College Station, Texas, USA; University of Georgia

**Keywords:** EV71, 3C protease, 4-iminooxazolidin-2-one

## Abstract

The fatal pathogen enterovirus 71 (EV71) is a major cause of hand-foot-and-mouth disease (HFMD), which leads to serious neurological syndromes. While there are no effective clinical agents available for EV71 treatment thus far, EV71 3C protease (3C^pro^), a cysteine protease encoded by the virus, has become a promising drug target for discovery of antiviral drugs, given that it plays a crucial role in virus proliferation and interferes with host cell function. Here, we report two inhibitors of EV71 3C^pro^, FOPMC and FIOMC, that were developed from previously reported cyanohydrin derivative (*R*)-1 by replacing the acyl cyanohydrin group with 4-iminooxazolidin-2-one. FOPMC and FIOMC have potent antiviral activity and dramatically improved metabolic stability. These two inhibitors demonstrated broad anti-EV effects on various cell lines and five epidemic viral strains. We further illuminated the binding models between 3C^pro^ and FOPMC/FIOMC through molecular docking and molecular dynamics simulations. The substitution of an acyl cyanohydrin group with 4-iminooxazolidin-2-one does make FOPMC and FIOMC potent anti-EV71 drug candidates as universal nonclassical bioisosteres with a cyanohydrin moiety.

**IMPORTANCE** EV71 is one of the most epidemic agents of HFMD. Thus far, there are no antiviral drugs available for clinical usage. The conserved EV71 3C^pro^ plays pivotal roles in virus proliferation and defense host immunity, as well as having no homology in host cells, making it a most promising antiviral target. In this work, we identified that propyl- and isopropyl-substituted 4-iminooxazolidin-2-one moieties (FOPMC and FIOMC) effectively inhibited five epidemic viral strains in rhabdomyosarcoma (RD), HEK-293T, and VeroE6 cell lines. The inhibition mechanism was also illustrated with molecular docking and molecular dynamics (MD) simulations. The successful replacement of the labile cyanohydrin greatly improved the stability and pharmacokinetic properties of (*R*)-1, making 4-iminooxazolidin-2-one a nonclassical bioisosteric moiety of cyanohydrin. This discovery addressed a critical issue of the primitive structural scaffold of these promising anti-EV71 inhibitors and could lead to their development as broad-spectrum anti-EV agents.

## INTRODUCTION

Hand-foot-and-mouth disease (HFMD) is a highly epidemic disease that predominantly affects infants (1). It is primarily caused by a class of enteroviruses that includes enterovirus 71 (EV71) and coxsackieviruses A16 and B3 (CVA16 and CVB3, respectively) (2). Among them, EV71 is the most fatal virus due to its neuroinvasiveness and was first isolated from sputum specimens of patients in California in 1969. Since then, it has become widespread around the world, especially in Asia and the Pacific. EV71 infection can lead to polio-like syndromes, including viral meningitis, encephalitis, myocarditis, pulmonary edema and paralysis, giving rise to a high mortality rate (3). In 2010, there was an outbreak of HFMD epidemic in China, affecting approximately 1.7 million people and resulting in 905 fatalities (4). In addition, a rare enterovirus, D68 (EV-D68), had rapid spread in the United States in 2014, which caused lower respiratory infections in 1,116 young children (5). However, no specific medications to clinically treat HFMD are available thus far (6).

EV71 belongs to the *Enterovirus* genus in the *Picornaviridae* family. The genome is a single-stranded and positive-sense RNA. The polyprotein precursor encoded by the RNA genome is further processed into VP1 to -4 of four structural proteins and 2A to -C and 3A to -D of seven nonstructural proteins (7, 8). EV71 3C^pro^ is a cysteine protease and cleaves the junction of the polyprotein, with the exception of VP1/2A by 2A protease (9). 3C^pro^ also plays a key role in the defense of the host’s innate immune and protein expression (10). Given the crucial roles in viral survival and propagation, 3C^pro^ could serve as a potential anti-EV71 drug target.

Several effective inhibitors of anti-EV71 3C^pro^ were generated through the structure-based design. Rupintrivirvr (AG7088), which originally served as a peptidomimetic inhibitor of human rhinovirus (HRV) 3C cysteine protease (11, 12), was able to effectively inhibit EV71 and EV-D68 replication (13, 14). Meanwhile, AG7088 analogues (AG7404 and SG85) were developed, which exhibited potent antirhinovirus and antienterovirus activity *in vitro* (15). Compared with HRV, AG7088 targeting EV71 demonstrated a significant decrease in potency (50% effective concentrations [EC_50_s] of 13 nM for HRV and 1.67 μM for EV71) (16–19). The crystal structure of EV71 3C^pro^ in complex with rupintrivir demonstrated that the S2 pocket of EV71 3C^pro^ featured a semiclosed state, and the S1′ pocket was too small to accommodate the P1′ unit of AG7088 (19, 20). Subsequently, a number of potential inhibitors have been exploited—for instance, substrate-based peptidomimetic derivatives and natural products (9). Previously, two 3C^pro^ inhibitors, NK-1.8k and NK-1.9k, were reported by our group to have significant inhibitory activity toward EV71 3C^pro^ at both enzymatic and cellular levels, thus effectively inhibiting EV71 infection (21, 22). In addition, some active molecules based on virtual screening and natural products could also serve as inhibitors of EV71 and EV-D68. The flavonoid natural product quercetin (EC_50_, 12.1 μM) effectively inhibited the enzyme activity of EV71 3C^pro^, blocking virus multiplication (23). Luteoloside (EC_50_, 0.43 mM) showed inhibition of cytopathy caused by EV71 infection, which was dose dependent *in vitro* (24). Fisetin (EC_50_, 85 μM) and rutin (EC_50_, 110 μM) also displayed a certain degree of anti-EV71 activity (25). In addition, pleconaril was reported to inhibit EV-D68 at an EC_50_ of 430 nM (26).

In 2015, a peptidomimetic compound, (*R*)-1, bearing a cyanohydrin warhead, was reported as a novel noncovalent inhibitor of EV71 3C^pro^ (27) (Table 1, compound 1). Despite the significant improvement in both selectivity and potency, the decay of the cyanohydrin group may lead to stability and toxicity issues (28). To overcome these deficiencies, we initially derivatized the hydroxyl group into esters and carbamates. After several rounds of modification, we observed the spontaneous autocyclization of N-monosubstituted carbamates yielded a 4-iminooxazolidin-2-one moiety, which greatly improved the chemical and metabolic stability and maintained comparable potency of (*R*)-1. Actually, 4-iminooxazolidin-2-one could apply as a nonclassical bioisosteric moiety of cyanohydrin. In the current work, we report that the propyl- and isopropyl-substituted 4-iminooxazolidin-2-one moieties (FOPMC and FIOMC) effectively restrained five enterovirus strains in rhabdomyosarcoma (RD), HEK-293T, and VeroE6 cell lines and displayed little cytotoxicity. Based on the docking models and molecular dynamics (MD) simulations, we further analyzed the molecular mechanism of FOPMC and FIOMC as 3C^pro^ inhibitors. In conclusion, the 4-iminooxazolidin-2-one moiety circumvented the disadvantages of cyanohydrin and could be applied as a nonclassical bioisostere to develop acylated cyanohydrin inhibitors for a wide range of cysteine proteases.

**TABLE 1:**
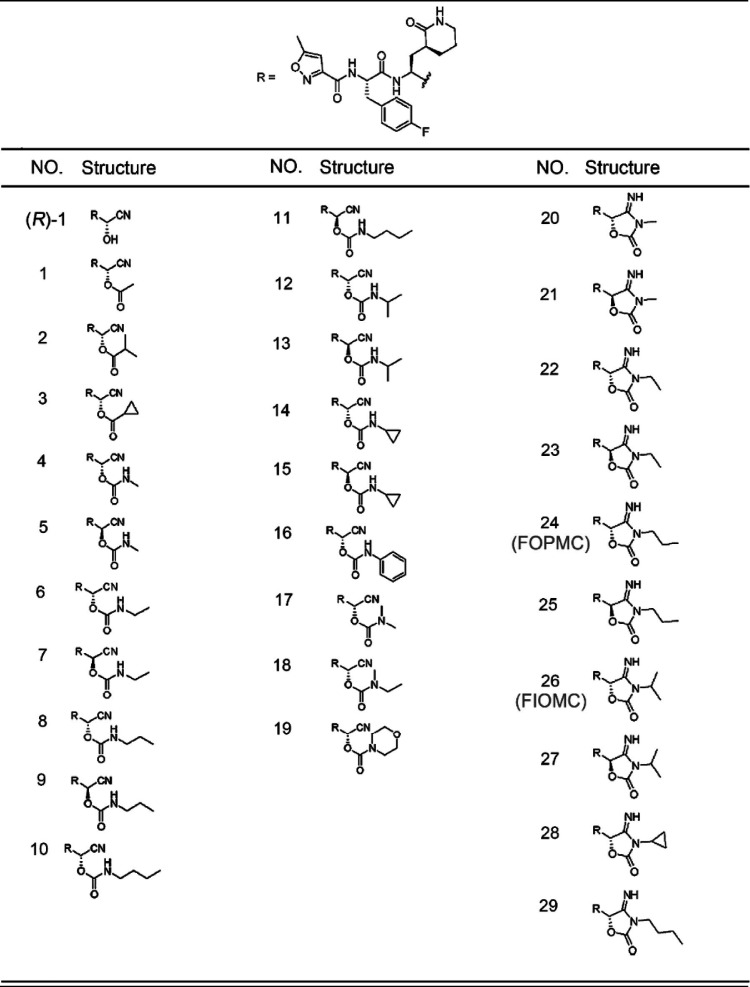
Inhibitors of 3C^PRO^

## RESULTS

### FOPMC and FIOMC inhibit EV71-GFP virus proliferation.

**FIG 1:**
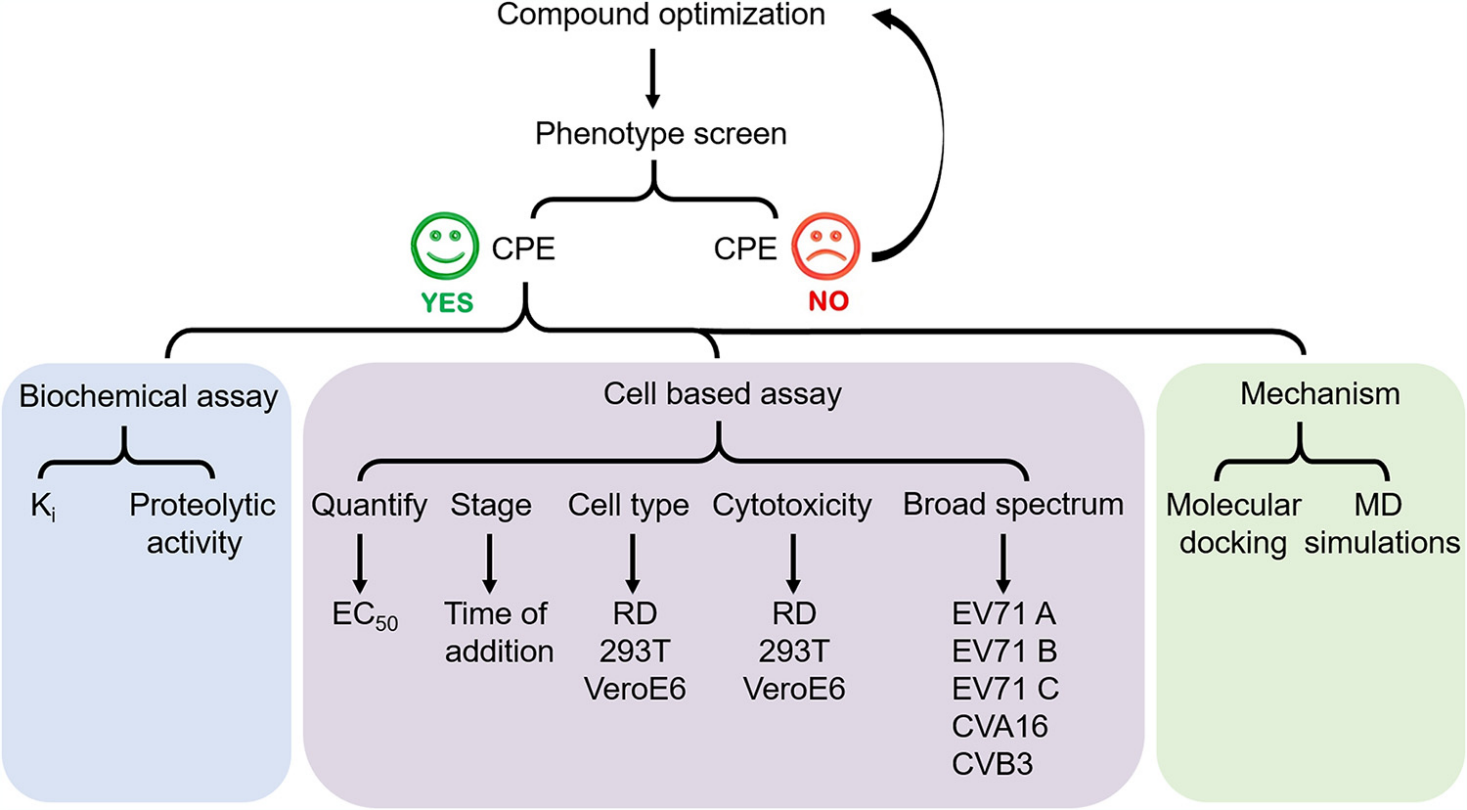
Schematic for the experimental procedure used in this study.

**FIG 2:**
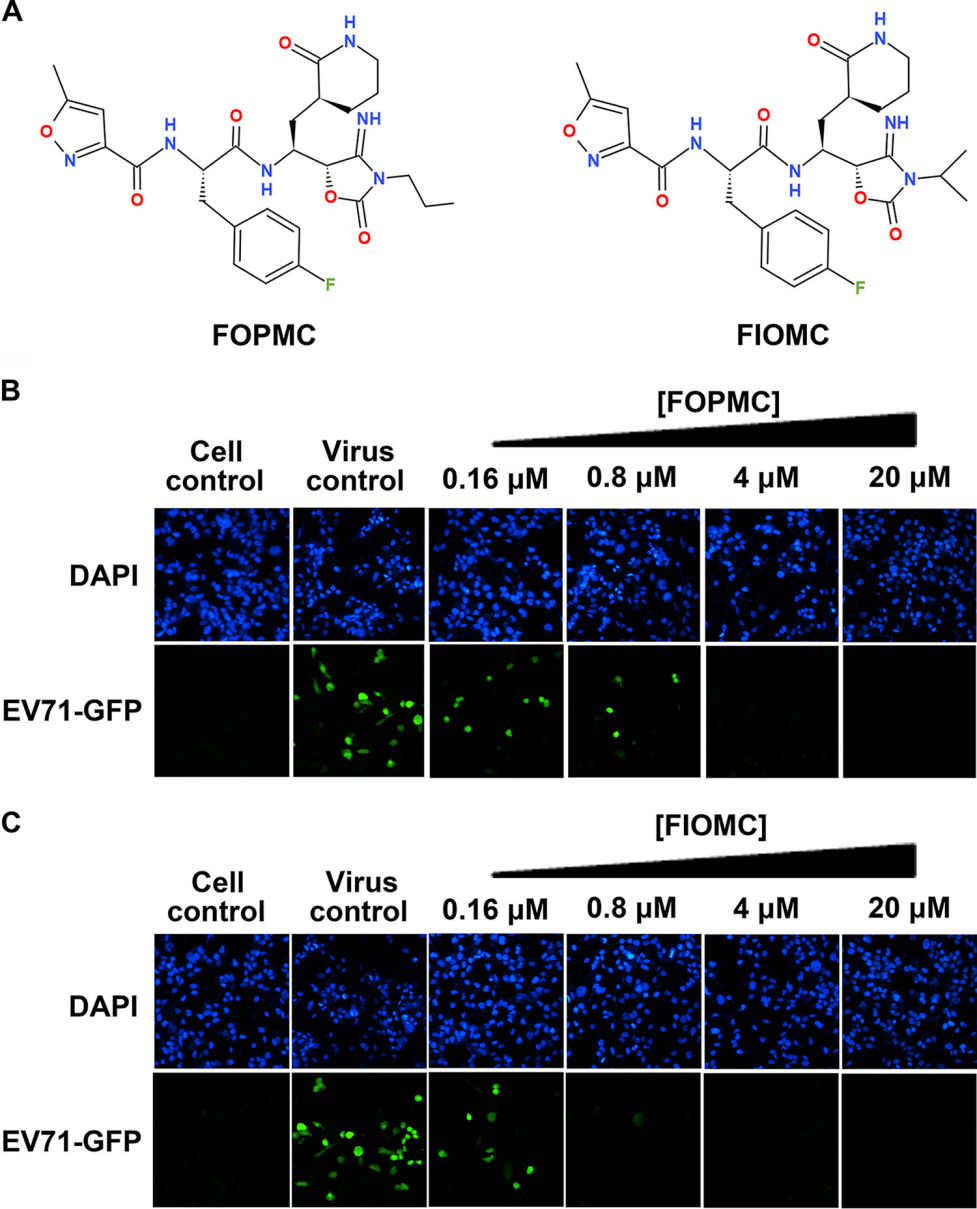
FOPMC and FIOMC effectively inhibited EV71 replication. (A) Chemical formulas of FOPMC and FIOMC. FOPMC (B) and FIOMC (C) inhibited EV71-GFP virus replication in RD cells in a concentration-dependent reduction manner. RD cells were treated with FOPMC and FIOMC (0.16 to 20 μM) and incubated with EV71-GFP for 24 h. The nucleus was stained with DAPI (4′,6-diamidino-2-phenylindole), as shown in the top panel. The GFP fluorescence signals are shown in the bottom panel.

(*R*)-1 was previously reported by our group as a novel anti-EV71 inhibitor with a cyanohydrin warhead and a fine-tuned peptidyl scaffold (27). This inhibitor significantly improved selectivity and antiviral activity. However, the cyanohydrin moiety raised potential toxicity and metabolic instability issues. To optimize stability and pharmacokinetic properties, we optimized the substituent groups on α-carbon and synthesized 29 new compounds based on (*R*)-1 (Table 1) (28). The schematic for the experimental procedure is represented in Fig. 1. The 4-iminooxazolidin-2-one derivatives FOPMC and FIOMC were discovered by modifying the labile cyanohydrin moiety (Fig. 2A). Both FOPMC and FIOMC showed positive results against replication of EV71 expressing green fluorescent protein (EV71-GFP) in a phenotype screen and exerted antiviral activity in a concentration-dependent manner, with a cytopathic effect (CPE) above 0.16 μM (Fig. 2B and C).

### FOPMC and FIOMC effectively inhibit EV71 proliferation.

**FIG 3:**
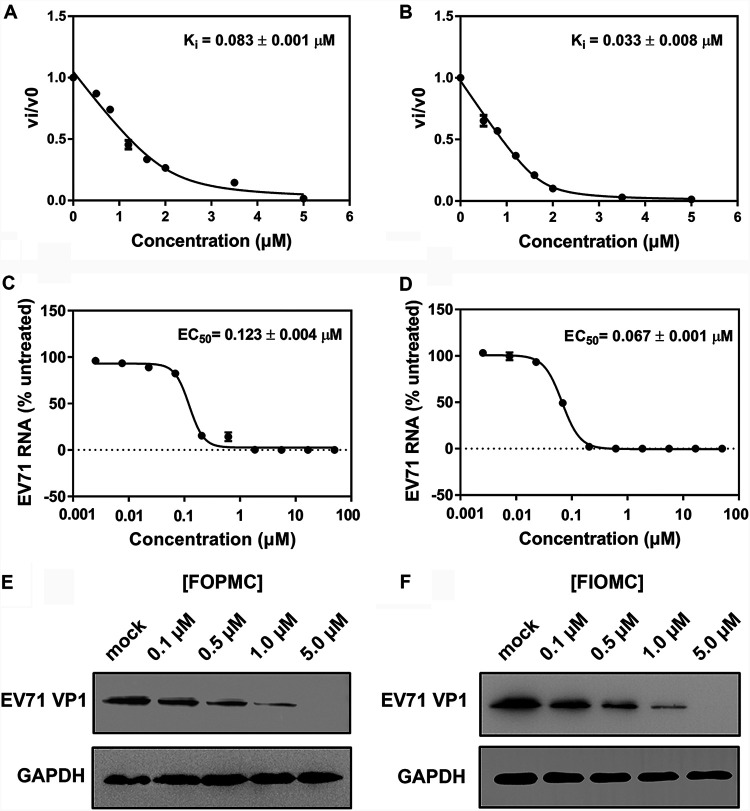
Quantification of the antiviral effects of FOPMC and FIOMC. (A to D) *K_i_*s of FOPMC (A) and FIOMC (B) and EC_50_s of FOPMC (C) and FIOMC (D). The levels of expression of EV71 VP1were inhibited by FOPMC (E) and FIOMC (F) in a dose-dependent reduction manner.

An inhibitor constant (*K_i_*) enzymatic assay was carried out to estimate the anti-EV71 3C^pro^ effects of FOPMC and FIOMC. The *K_i_*s of FOPMC and FIOMC were 0.083 ± 0.001 and 0.033 ± 0.008 μM, respectively (Fig. 3A and B). The anti-EV71 activities of FOPMC and FIOMC were further investigated by quantitative assays using EV71 Fuyang virus. The EC_50_s of FOPMC and FIOMC was 0.123 ± 0.004 and 0.067 ± 0.001 μM, respectively, which represented concentration-dependent decline models (Fig. 3C and D). In addition, we characterized the expression of EV71 VP1, which was restrained with the treatment of inhibitors, while the expression of glyceraldehyde-3-phosphate dehydrogenase (GAPDH) was invariable (Fig. 3E and F). Together, the results consolidated that FOPMC and FIOMC inhibit EV71 proliferation in RD cells.

### FOPMC and FIOMC impact on EV71 viral replication stage.

To explore which infection stages were impacted by the compounds, time-of-addition assays were conducted. In this case, the single-round EV71 luciferase-expressing virus was used to test virus propagation when treated with FOPMC and FIOMC, which was beneficial to exclude reinfection with the virus. NK-1.8k targeting EV71 3C^pro^ and GPP3 inhibiting virus entry were applied as controls (29).

**FIG 4:**
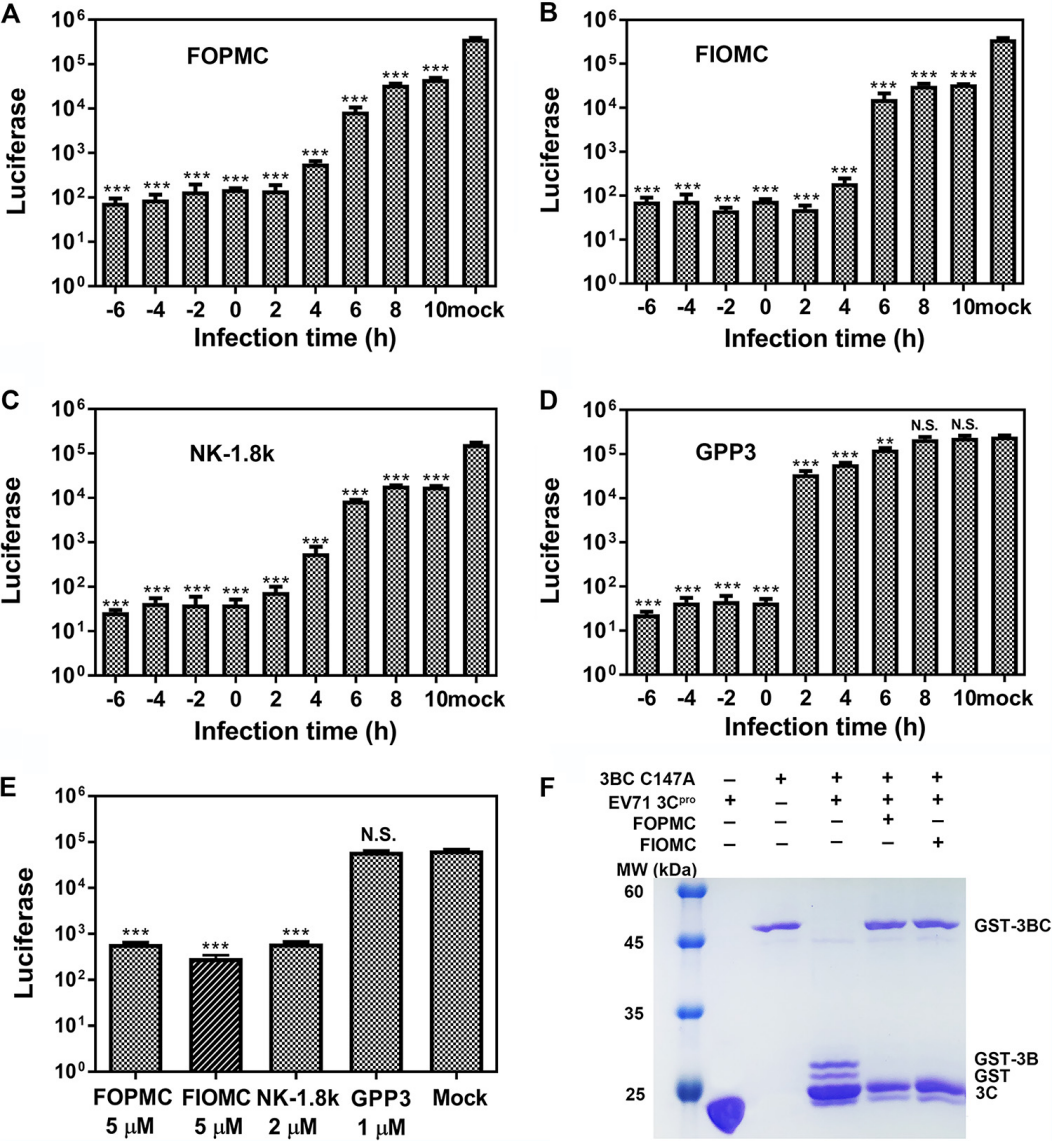
FOPMC and FIOMC play inhibitory roles in the viral replication stage. RD cells were treated with FOPMC (A) or FIOMC (B), NK-1.8k (C), or GPP3 (D) and incubated with EV71-luciferase virus at −6, −4, −2, 0, 2, 4, 6, 8, and 10 hpi. (E) RD cells were treated with inhibitors and pEV71-replicon RNA. (F) Inhibitory activity of FOPMC and FIOMC against EV71 3C^pro^ to hydrolyze the precursor 3BC. Statistical significance was evaluated by *t* test. **, *P* < 0.01; ***, *P* < 0.001; N.S., not significant.

From −6 to 10 h postinfection (hpi), both FOPMC and FIOMC exhibited valid inhibitory effects regardless of the time (Fig. 4A and B), which was similar to the results of the virus replication inhibitor NK-1.8k (Fig. 4C). Different from the above results, the antiviral effect of the virus entry inhibitor GPP3 was dramatically decreased from 4 hpi (Fig. 4D). What’s more, the replicon results also indicated that FOPMC and FIOMC impacted the virus replication stage (Fig. 4E).

We further evaluated the inhibitory activity of FOPMC and FIOMC against EV71 3C^pro^ to hydrolyze the precursor protein. EV71 3C^pro^ was treated with FOPMC and FIOMC and incubated with polyprotein 3BC-C147A. The SDS-PAGE results suggested that FOPMC and FIOMC distinctly restrained the hydrolysis of the 3C^pro^ on 3BC (Fig. 4F). All the results inidicated that FOPMC and FIOMC suppressed viral reproduction by specifically inhibiting the hydrolysis of 3C^pro^ on EV71 polyproteins.

### FOPMC and FIOMC suppress EV71 proliferation on various cell lines.

**FIG 5:**
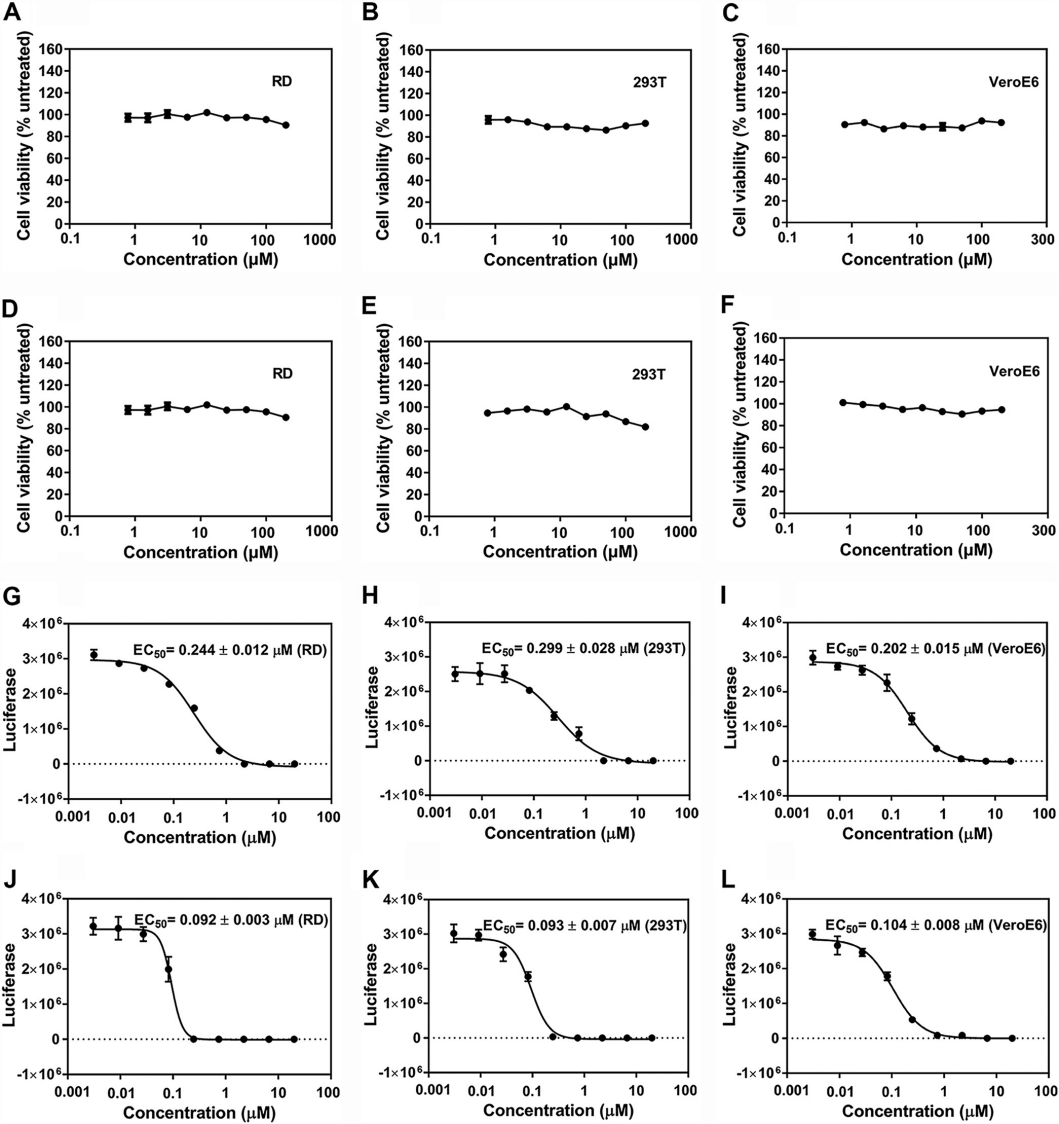
Cytotoxicity and EC_50_s of FOPMC and FIOMC for various cell lines. (A to F) Cytotoxicity of FOPMC (A, B, and C) and FIOMC (D, E, and F) for RD, HEK-293T, and VeroE6 cells. (G to L) EC_50_s of FOPMC (G, H, and I) or FIOMC (J, K, and L) for RD, HEK-293T, and VeroE6 cells. All assays were repeated three times.

To evaluate whether the cell types or species affected the abilities of FOPMC and FIOMC to inhibit EV71 virus, we first tested the cytotoxicity of FOPMC and FIOMC on RD, HEK-293T, and VeroE6 cells. Even at 200 μM, both FOPMC (Fig. 5A to C) and FIOMC (Fig. 5D to F) showed no apparent cytotoxic effect on three different cell lines.

In addition, we measured the antiviral activity on various cell lines. The EC_50_s of FOPMC were 0.244 ± 0.012 μM for RD cells, 0.299 ± 0.028 μM for HEK-293T cells, and 0.202 ± 0.015 μM for VeroE6 cells, respectively (Fig. 5G to I). Correspondingly, the EC_50_s of FIOMC were 0.092 ± 0.003 μM for RD cells, 0.093 ± 0.007 μM for HEK-293T cells, and 0.104 ± 0.008 μM for VeroE6 cells, respectively (Fig. 5J to L). Taken together, FOPMC and FIOMC could potently inhibit EV71 proliferation on various cells.

### FOPMC and FIOMC are broad anti-EV inhibitors.

**FIG 6:**
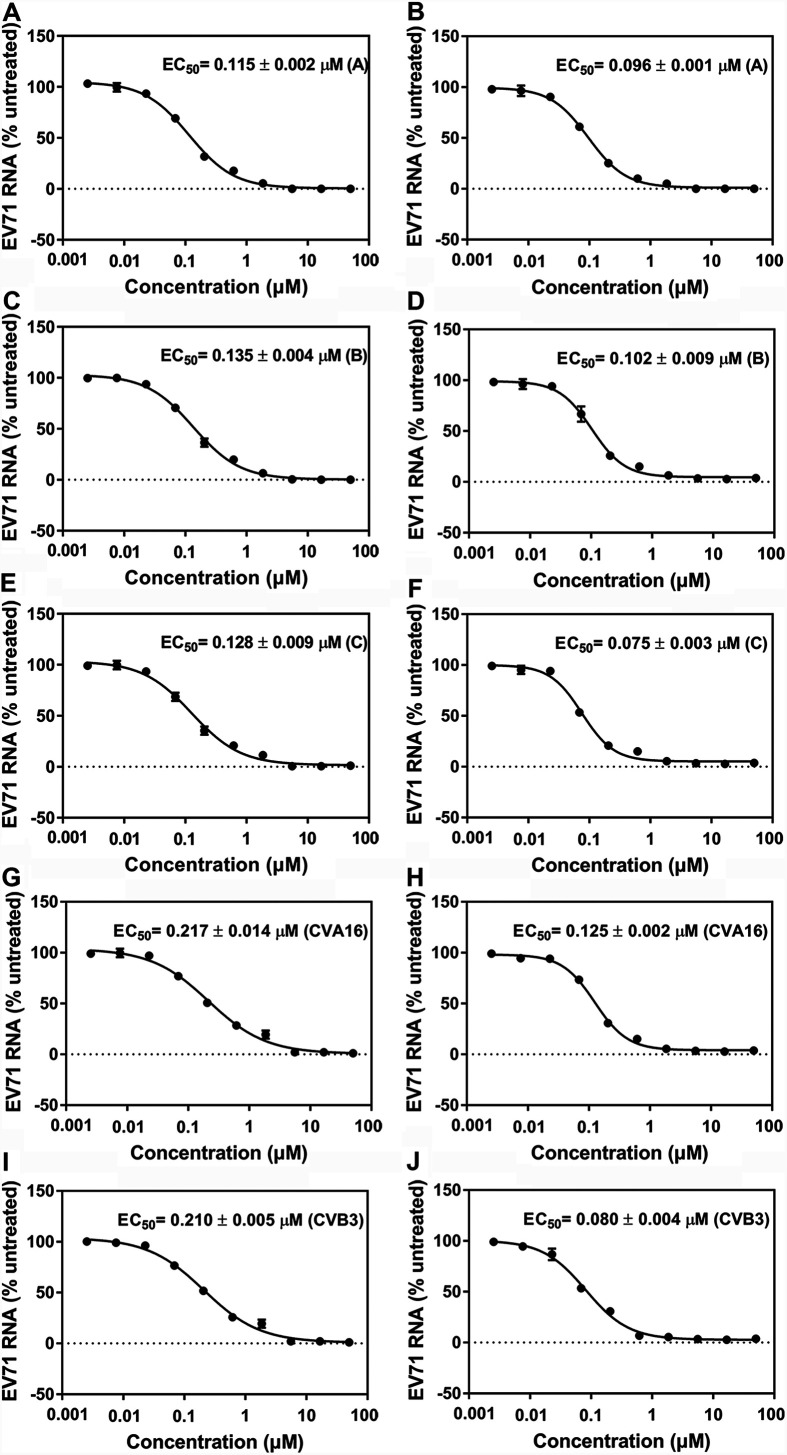
Antiviral activity of FOPMC and FIOMC on five virus strains. Shown are the antiviral activities of FOPMC (A, C, E, G, and I) and FIOMC (B, D, F, H, and J). The EC_50_ of antiviral activity was determined by qRT-PCR.

Multiple enteroviruses, including EV71 (types A, B, and C), CA16, and CVB3, are associated with HFMD diseases (30). We detected the antiviral spectrums of FOPMC and FIOMC by infecting RD cells with five virus strains. The EC_50_s of FOPMC on RD cells were 0.115 ± 0.002 μM (EV71-A), 0.135 ± 0.004 μM (EV71-B), 0.128 ± 0.009 μM (EV71-C), 0.217 ± 0.014 μM (CVA16), and 0.210 ± 0.005 μM (CVB3), respectively (Fig. 6A, C, E, G, and I). The EC_50_s of FIOMC were 0.096 ± 0.001 μM (EV71-A), 0.102 ± 0.009 μM (EV71-B), 0.075 ± 0.003 μM (EV71-C), 0.125 ± 0.002 μM (CVA16), and 0.080 ± 0.004 μM (CVB3), respectively (Fig. 6B, D, F, H, and J). Overall, both FOPMC and FIOMC could serve as broad-spectrum anti-EV agents to treat HFMD. As determined in the broad infection assays, FIOMC consistently demonstrated higher potency as the leading compound.

### Molecular mechanism of FOPMC and FIOMC as EV71 3C^pro^ inhibitors.

**FIG 7:**
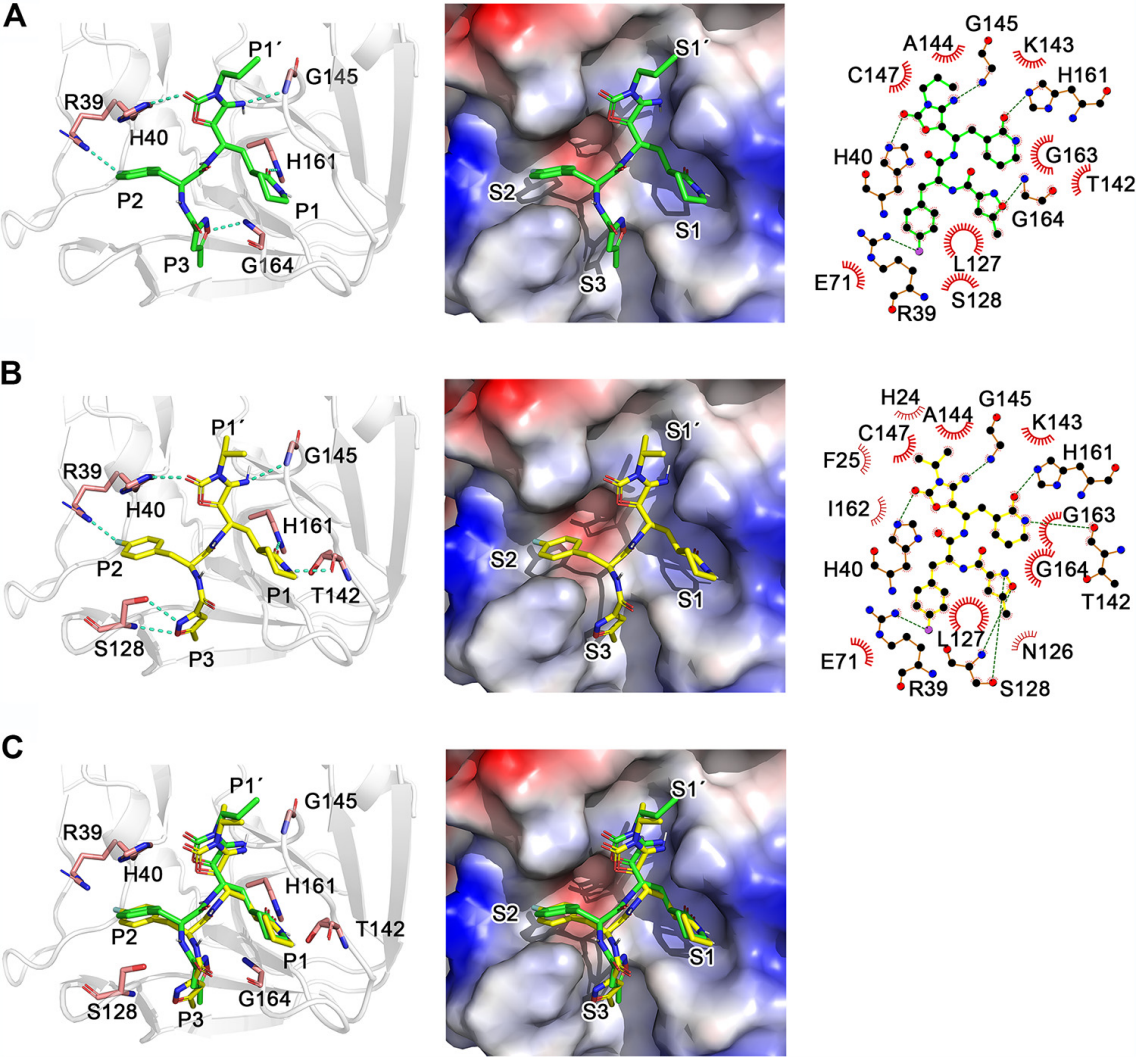
Molecular docking models of FOPMC, FIOMC, and EV71 3C^pro^. (A and B) Binding models of FOPMC (A) and FIOMC (B) with EV71 3C^pro^ (PDB accession no. 5BPE). (C) The superimpositions of FOPMC and FIOMC are shown in identical orientations. The EV71 3C^pro^ model is shown as a cartoon, and FOPMC (green), FIOMC (yellow), and catalytic triad residues H40, E71, C147 (pink) are shown as sticks (left). EV71 3C^pro^ electrostatic potential surfaces are illustrated in the middle, and interactions between two inhibitors and 3C^pro^ are illustrated on the right. Schematics were generated by LIGPLOT + 1.4. The hydrophobic contacts are indicated as an “eyelash” motif. Hydrogen bonds are indicated as green dashed lines. S1′, S1, S2, and S3 are represented for substrate binding pockets.

To illustrate the inhibitory mechanism, we utilized the AutoDock Vina program to dock FOPMC and FIOMC into the EV71 3C^pro^ crystal structure. The two inhibitors were noncovalently bound to 3C^pro^ and coordinated by the circumambient amino acid residues through hydrophobic and polar interactions (Fig. 7A to C). The S1 pocket was constituted by T142, H161, G163, and G164. The (*S*)-δ-lactam of FIOMC formed two hydrogen bonds with the side chains of T142 and H161. In contrast, FOPMC failed at forming a hydrogen bond with T142, except with H161. The S2 pocket was capable of accommodating the more deeply sunk P2 *para*-fluorine benzyl group. R39 and H40 were located at the back of the S2 pocket, both forming polar interactions with two inhibitors. The P3 methylisoxazole group extended vertically along the binding groove and occupied the S3 pocket. G164, on the edge of the S3 pocket, formed a hydrogen bond with FOPMC, while Ser128, on the other side of this pocket, produced stronger polar interactions with FIOMC, generating two hydrogen bonds with distances of 2.8 and 2.9 Å, respectively. The cyanohydrin occupied the S1′ pocket and closely interacted with G145 in both inhibitors.

**FIG 8:**
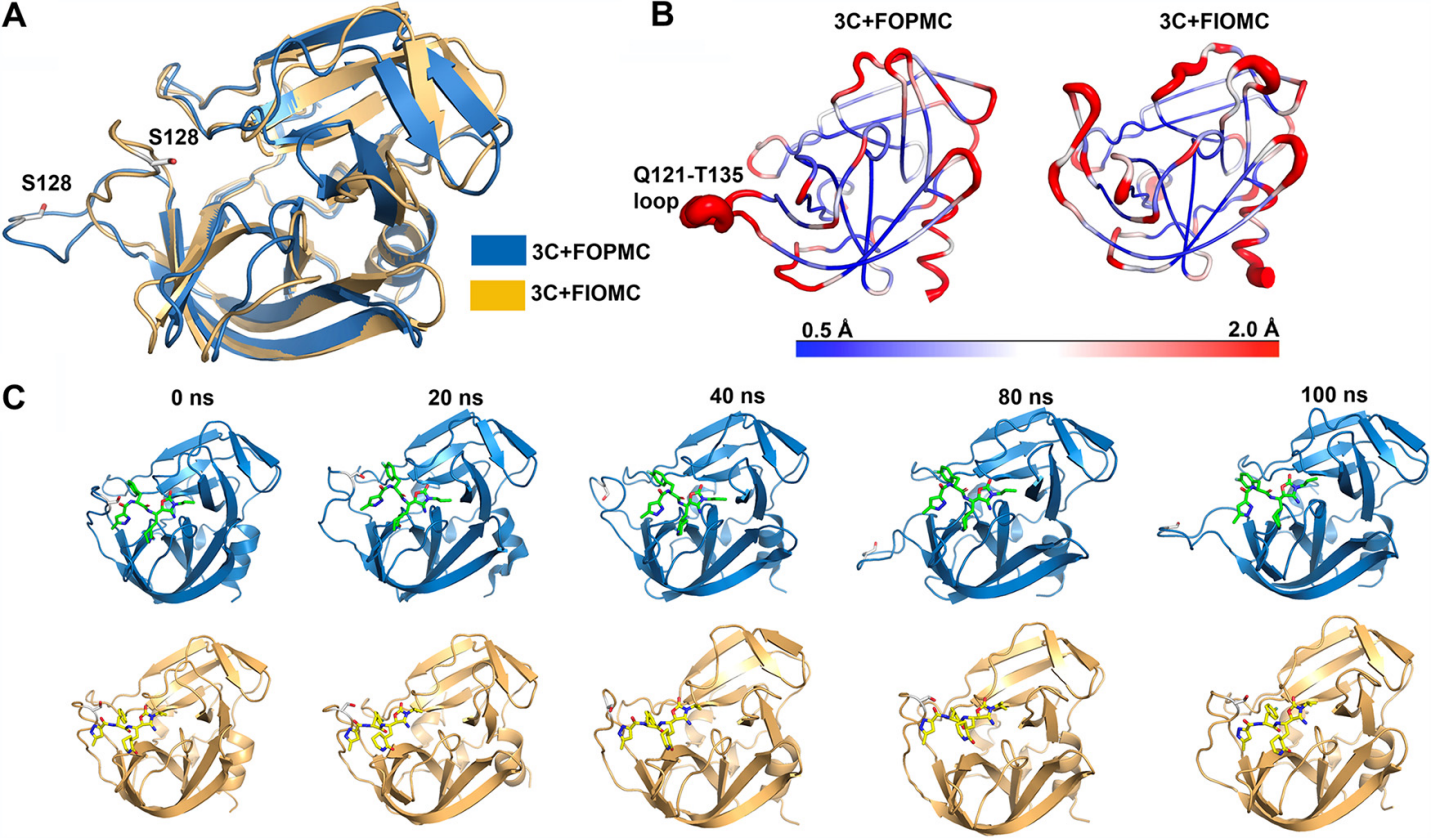
MD equilibrated conformations of EV71 3C^pro^-FOPMC and 3C^pro^-FIOMC complexes. (A) Superimposed structural models of 3C^pro^ when interacting with FOPMC (blue) or FIOMC (orange). The S128 residue in the Q121-T135 loop region is highlighted as sticks. (B) The Q121-T135 loop was more flexible in the 3C^pro^-FOPMC complex than that in the 3C^pro^-FIOMC complex, as determined by root mean square fluctuation (RMSF) analysis (where red indicates flexible regions, and blue indicates rigid regions). (C) Snapshots of protein-ligand conformations throughout the MD trajectories (green, FOPMC; yellow:, FIOMC). At approximately 40 ns, the Q121-T135 loop flipped away from FOPMC due to the absence of polar interactions between S128 and FOPMC. In contrast, S128 engaged with FIOMC during the entire simulations.

We further investigated the dynamic features of EV71 3C^pro^ upon binding to FOPMC and FIOMC, respectively (Fig. 8). The inhibitor FIOMC resided in the pocket composed of R39, H161, T142, S168, etc., residues throughout the MD simulation (see Movie S1 in the supplemental material), while FOPMC deviated from the original binding pose (see Movie S2 in the supplemental material). Notably, the loop region (residues Q121 to T135) flipped away from the binding pocket in the 3C^pro^-FOPMC complex, indicating that the lacking of polar interactions between S128 and the isoxazole ring reduced the affinity of FOPMC at the P3 site. Indeed, an estimation of binding energies through MM-PBSA (i.e., molecular mechanics Poisson-Boltzmann surface area) calculations suggested that the binding capacity of FIOMC (binding energy of −44.3 ± 1.2 kJ/mol) was significantly higher than that of FOPMC (binding energy of −22.9 ± 1.7 kJ/mol), which corroborated our experimental measurements (Fig. 3, 5, and 6).

The molecular docking models and MD simulations results elucidated the detailed mechanism of the interaction between inhibitors and 3C^pro^ at the molecular level and also explained why FIOMC had a better antiviral effect than FOPMC. Although we merely optimized the acyl cyanohydrin in the P1′ position, it apparently affected the interaction between inhibitors and the entire binding pocket. These results indicated that the P1′ position was crucial for the design of inhibitors of EV71 3C^pro^.

## DISCUSSION

As a reliable strategy for the discovery of novel inhibitors and for refining the pharmacological properties of existing drugs, bioisosterism has been successfully applied in the pharmaceutical industry over the years (31). Bioisosterically related pairs include both natural products and synthetic compounds, such as γ-aminobutyric acid (GABA) and muscimol (32), neurotransmitters glutamate and AMPA (α-amino-hydroxy-5-methyl-4-isoxazolepropionic acid) (33), hydroxyisooxazole, and members of the carboxyl group (31). In the current work, we explored the bioisosteric relationship between cyanohydrin and 4-iminooxazolidin-2-one with the aim of reducing adverse effects and enhancing stability for one of the most potent EV71 inhibitors, (*R*)-1. The cyanohydrin warhead of (*R*)-1 released cyanide upon hydrolysis, leading to inherent stability (half-life [*t*_1/2_] in human plasma of 11 min) and potential toxicity problems. By replacing the cyanohydrin group with 4-iminooxazolidin-2-one, we obtained two new compounds, FOPMC and FIOMC, the *t*_1/2_ of which exceed 120 min in both human and mouse plasma (28). Given the high propensity of the mutational potential of RNA viruses, we selected viruses resistant to FOPMC and FIOMC by continuously culturing EV71 for 20 rounds. Sequence analyses showed no mutation of EV71 virus: thus, these two inhibitors could serve as long-lasting treatment agents. More importantly, no apparent cytotoxicity was observed for FOPMC or FIMOC in the three cell lines we tested.

As analogues to the previously reported (*R*)-1 molecule, FOPMC and FIOMC inherited its potency and selectivity toward EV71 3C^pro^. We further verified their broad effects on five viral strains. Their abilities to inhibit viral replication were consistent, with FIOMC exhibiting higher potency (EC_50_s of 80 to 125 nM, depending on the specific virus). In summary, the 4-iminooxazolidin-2-one moiety maintained the effective antiviral activity of (*R*)-1 and improved its stability more than 10 times. The substitution of cyanohydrin with 4-iminooxazolidin-2-one provides the possibility to develop a broad spectrum of EV inhibitors with potential activity against other viral 3C proteases.

## MATERIALS AND METHODS

### EV71-GFP and luciferase reporter virus.

EV71 reporter virus expressing green fluorescent protein (GFP) or luciferase was prepared as previously reported (34). Briefly, the linearized EV71-GFP plasmid was transcribed into RNA and then transfected into HEK-293T cells. The pEV71-capsid plasmid was transfected into HEK-293T cells, and cells were cultured overnight. Subsequently, the pEV71-replicon RNA was transfected into the same cells. All of the cell supernatants were centrifuged, and the virus titrations were determined on human rhabdomyosarcoma (RD) cells.

### Virus titration on RD cells.

One hundred microliters of RD cells (3 × 10^4^ per well) was cultured for 24 h. EV71-A, -B, and -C, CVA16, and CVB3 were serially diluted from10^−1^ to 10^−9^ and added to RD cells, respectively. After 3 days, the titers of virus were calculated by endpoint dilution assays (EPDAs). All of the cells were infected with different viruses at a multiplicity of infection (MOI) of 1 in the subsequent experiments.

### Phenotype assay of inhibitors.

RD cells (3 × 10^4^ per well) were cultured in 96-well plates overnight. Diluted compounds (0.16 to 20 μM) and EV71-GFP reporter virus were added to RD cells. After 24 hpi, the GFP was monitored using confocal microscopy (Olympus, Japan).

### Expression and purification of 3C and 3BC proteins.

The EV71 3C^pro^ plasmid was amplified and linked into a pET-28a vector by NcoI and XhoI with a 6×His tag for protein overexpression and purification. Escherichia coli cells were induced at an optical density at 600 nm (OD_600_) of 0.8 and cultured at 18°C for 16 h. The collected cell pellet was resuspended in 25 mM Tris-HCl–300 mM NaCl (pH 7.5) and ultrasonically homogenized (Xinzhi, China). The supernatants, containing EV71 3C^pro^, were mixed with Ni beads. The nonspecific proteins were eluted with 50 mM imidazole. The 3C^pro^ was eluted with 300 mM imidazole and further separated by a HiTrap S ion exchange column. The peak of 3C^pro^ was collected for further analysis.

The precursor protein 3BC, with a C147A mutation, was cloned into the pGEX-6p-1 vector. Protein expression and purification were performed according to the method described above, with some modifications. The protein was cultured at 16°C and purified with glutathione-Sepharose 4B (GE Healthcare, USA).

### Determination of the inhibitor constants (*K_i_*) of FOPMC and FIOMC.

The inhibitor constants (*K_i_*s) were determined by fluorescence resonance energy transfer (FRET) using NMA-IEALFQGPPK(DNP)FR peptide. The assays were performed with 50 mM Tris-HCl–150 mM NaCl (pH 7.0) with 0.5 μM EV71 3C^pro^ and gradient-diluted FOPMC/FIOMC. Subsequently, 100 μM each peptide was hydrolyzed for 2 h. The fluorescence signals were monitored at a microplate reader (Perkin Elmer, USA).

### qRT-PCR of FOPMC and FIOMC.

The viral genome replication inhibition in host cells after treatment with FOPMC and FIOMC was monitored by EV71 genotype C Fuyang virus. Briefly, RD cells were cultured at 1.5 × 10^5^ per well at 37°C for 24 h and then incubated with gradient-diluted FOPMC, FIOMC, and virus. The intracellular RNAs were extracted using TRIzol (TransGen, China) after 24 hpi. The real-time quantitative PCR (qRT-PCR) assays were performed with a SYBR RT-PCR kit (Bio-Rad, USA) for the EV71 5′ untranscribed region (UTR) and the host GAPDH. The transcript copies of EV71 5′ UTR and GAPDH were calculated using the threshold cycle (ΔΔ*C_T_*) method (35).

### Time-of-addition assay of FOPMC and FIOMC.

We conducted the time addition assay with FOPMC, FIOMC, NK-1.8k, and an GPP3 to explore the infection stage at which the compounds demonstrated inhibitory effects. RD cells (3 × 10^4^ per well) were treated with FOPMC (5 μM), FIOMC (5 μM), GPP3 (1 μM), NK-1.8k (2 μM), and EV71-luciferase virus, respectively. Another plate of RD cells was treated with the same concentrations of the four inhibitors and transfected with pEV71-replicon RNA. After 24 hpi, the luciferase activity was determined using Bright-Glo luciferase substrate (Promega, USA).

### Hydrolytic activity inhibition of EV71 3C^pro^.

To ascertain whether FOPMC and FIOMC inactivated EV71 3C^pro^ to hydrolyze the precursor 3BC, the fusion protein GST-3BC with a catalytic central residue C147A mutation was expressed. A 100 μM concentration of EV71 3C^pro^ and FOPMC/FIOMC (at a molar ratio of 1:5) was incubated at 30°C for 2 h. GST-3BC (50 μM) protein was added for another 4 h, and the reagents were analyzed using SDS-PAGE.

### EC_50_s of FOPMC and FIOMC on different cell lines.

One hundred microliters each of RD (3 × 10^5^ cells/ml), VeroE6 (3 × 10^5^ cells/ml), and HEK-293T cells (2 × 10^5^ cells/ml) was cultured overnight. Each 96-well plate of cells was treated with EV71-luciferase virus and diluted FOPMC/FIOMC (diluted from 3 nM to 20 μM) for 24 h. The luciferase signals were tested using the firefly luciferase kit (Promega, USA). All of the EC_50_s were profiled by Graph Pad Prism 7.0.

### Cytotoxicity of FOPMC and FIOMC.

Determination of the cytotoxicity of FOPMC and FIOMC was performed on three cell lines using a CellTiter-Glo kit (Promega, USA). Briefly, RD, VeroE6, and HEK-293T cells were treated with gradient dilutions of FOPMC/FIOMC (from 0.78 to 200 μM) for 48 h. CellTiter-Glo reagents were applied to evaluate the cells’ viability by a microplate reader.

### Antiviral activity of FOPMC and FIOMC.

To evaluate the antiviral effect of FOPMC and FIOMC on various viral strains, we measured the EC_50_s of these two inhibitors on five virus strains. RD cells were treated with FOPMC/FIOMC (0.003 to 50 μM) and each viral strain for 2 h. The antiviral activity was conducted by qRT-PCR according to the method described above.

### Molecular docking.

The molecular interactions between FOPMC, FIOMC, and EV71 3C^pro^ were predicted by the AutoDock Vina program. The crystal structures of EV71 3C^pro^ and (*R*)-1 were used as the initial model (PDB accession no. 5BPE). FOPMC and FIOMC were built and optimized to the local energy minima. The bound ligands were deleted from the PDB file. The side chains of the residues within 4 Å of the ligand were treated as flexible during docking.

### Molecular dynamics simulation.

The 100-ns all-atom simulations performed for the complex of 3C^pro^ and compounds were performed with the Gromacs 2019.6 package (36, 37). The initial poses of inhibitor-binding conformations were adopted from the docking results. The system was solvated in a box (43.3 by 38.7 by 41.9 Å^3^) with TIP3P waters and 0.15 M NaCl of about 26,286 atoms in total. The CHARMM27 force field was adopted (38), wherein the topologies of inhibitors were generated by the SwissParam server (39). First, the energy minimizations were performed to relieve unfavorable contacts, followed by 10-s equilibration steps. Subsequently, a simulated-annealing procedure was applied, which raised the temperatures to 500 K in 5 ns followed by slowly cooling down to 300 K. Then the product run was performed under conditions of a velocity-rescale thermostat (300 K) and Parrinello-Rahman NPT ensemble. A 10-Å cutoff was set for nonbonded interactions, and the particle mesh Ewald (PME) method was used for electrostatic calculations. LINCS constraints were applied to H-bonds, and the time step was 2 fs. The binding conformations were clustered based on root mean square deviation (RMSD) calculations, and g_mmpbsa modules were evoked for the calculation of binding energies between the inhibitors and protein (40).
