# Supplementary material for: 4-Iminooxazolidin-2-One as a Bioisostere of Cyanohydrin Suppresses EV71 Proliferation by Targeting 3Cpro
Source: Microbiol Spectr. 2021 Nov 17;9(3):e01025-21. doi: 10.1128/Spectrum.01025-21 (PMC8597634; doi:10.1128/Spectrum.01025-21)
Supplement: SUPPLEMENTAL FILE 1 — Supplemental material. Download SPECTRUM01025-21_Supp_1_seq10.pdf, PDF file, 0.3 MB [file spectrum01025-21_supp_1_seq10.pdf]

Supplementary Material

4-Iminooxazolidin-2-one as a bioisostere of cyanohydrin suppresses EV71 proliferation by targeting 3C^{pro}

Binghong Xu[a, §], Meijun Liu[a, §], Sen Ma[a], Yuying Ma[c], Si Liu[a], Luqing Shang[b], Cheng Zhu[a,]*, Sheng Ye[a,]*, Yaxin Wang[a,]*

[a] Tianjin Key Laboratory of Function and Application of Biological Macromolecular Structures, School of Life sciences, Tianjin University, 92 Weijin Road, Nankai District, Tianjin 300072, P.R. China;

[b] College of Pharmacy, Nankai University, 38 Tongyan Road, Jinnan District, Tianjin 300353, P.R. China;

[c] Department of Chemistry, Texas A&M University, College Station, Texas 77843, United States.

*Corresponding authors:

Cheng Zhu, School of Life sciences, Tianjin University, Tianjin 300072, China, Email: cheng_zhu@tju.edu.cn;

Sheng Ye, School of Life sciences, Tianjin University, Tianjin 300072, China, Email: sye@tju.edu.cn;

Yaxin Wang, School of Life sciences, Tianjin University, Tianjin 300072, China, Email: wangyaxin@tju.edu.cn;

[§] These authors contributed equally to the work

Figure Legends

Supplementary movie 1： The dynamic trajectory of EV71 3C^{pro} interacted with FOPMC. The EV71 3C^{pro} was shown in cartoon (blue) and FOPMC was shown in sticks (green).

Supplementary movie 2： The dynamic trajectory of EV71 3C^{pro} interacted with FIOMC. The EV71 3C^{pro} was shown in cartoon (orange) and FIOMC was shown in sticks (yellow).
